# 'Cold' crystallization in nanostructurized 80GeSe_2_-20Ga_2_Se_3_ glass

**DOI:** 10.1186/s11671-015-0775-9

**Published:** 2015-02-06

**Authors:** Halyna Klym, Adam Ingram, Oleh Shpotyuk, Laurent Calvez, Elena Petracovschi, Bohdan Kulyk, Roman Serkiz, Roman Szatanik

**Affiliations:** Lviv Polytechnic National University, 12 Bandera str, Lviv, 79013 Ukraine; Physics Faculty of Opole University of Technology, 75 Ozimska str, Opole, 45370 Poland; Lviv Institute of Materials of SRC ‘Carat’, 202 Stryjska str, Lviv, 79031 Ukraine; Institute of Physics of Jan Dlugosz University, 13/15 al. Armii Krajowej, Czestochowa, 42201 Poland; Equipe Verres et et Céramiques, UMR-CNRS 6226, Institute des Sciences chimiques de Rennes, Université de Rennes 1, Rennes, Cedex 35042 France; Ivan Franko National University of Lviv, 1 Universytetska str, Lviv, 79000 Ukraine; Opole University, 11a Kopernika sq, Opole, 45040 Poland

**Keywords:** Chalcogenide glass, Crystallization, Annealing, Positron annihilation, Trapping

## Abstract

'Cold' crystallization in 80GeSe_2_-20Ga_2_Se_3_ chalcogenide glass nanostructurized due to thermal annealing at 380°C for 10, 25, 50, 80, and 100 h are probed with X-ray diffraction, atomic force, and scanning electron microscopy, as well as positron annihilation spectroscopy performed in positron annihilation lifetime and Doppler broadening of annihilation line modes. It is shown that changes in defect-related component in the fit of experimental positron lifetime spectra for nanocrystallized glasses testify in favor of structural fragmentation of larger free-volume entities into smaller ones. Nanocrystallites of Ga_2_Se_3_ and/or GeGa_4_Se_8_ phases and prevalent GeSe_2_ phase extracted mainly at the surface of thermally treated samples with preceding nucleation and void agglomeration in the initial stage of annealing are characteristic features of cold crystallization.

## Background

The Se-based chalcogenide glasses (ChGs) possessing good transparency in 0.8 to 16 μm spectral range are widely used in optoelectronic systems exploring thermal and optical imaging effects in both atmospheric telecommunication windows (3 to 5 and 8 to 12 μm) [1,2]. They also possess an excellent glass-forming ability, mechanical and chemical stability, which makes them one of the most unprecedented media for different IR fiber-optic applications [3,4]. It is known that crystallization of such ChG can improve their physical, mechanical, and thermal properties considerably, but it is difficult to produce IR-transmitting glass-ceramics properly because growing crystals is generally out of control during heat treatment, which makes the material opaque [5,6].

Such crystallization processes can be adequately studied at the level of *atomistic structural arrangement* using numerous experimental measuring techniques, such as IR vibrational and Raman scattering spectroscopy, X-ray diffraction (XRD), X-ray photoelectron spectroscopy, atomic force microscopy (AFM) and scanning electron microscopy (SEM), nuclear magnetic resonance, etc. [7-12]. However, the row of experimental probes available to study *atomic-deficient void structure* of such materials is rather limited, especially at nanometer and sub-nanometer scale. One of the best techniques capable to identify such finest free-volume voids is positron annihilation lifetime (PAL) spectroscopy, the method grounded on physical phenomenon of electron interaction with its antiparticle (positron) in a matter [13-15]. In application to semiconductor materials, this method is used to identify intrinsic free volumes owing to simple models considering competitive channels of positron trapping from delocalized defect-free bulk states, deep ground states of positron traps (extended free-volume defects), and decaying of bounded positron-electron (positronium (Ps)) states [13,16]. In the measuring mode of Doppler broadening of annihilation line (DBAL), this technique allows additional identification of dominant positron trapping sites in the tested objects [13,17]. So, combined PAL-DBAL measurements are expected to be useful to study atomic-deficient void structure of solids affected by different nanosctructurization treatments, in part those producing nanosized inhomogeneities like extractions of segregated inner phases, nucleates, agglomerates, and fragments of crystallites, vacancy clusters and free-volume voids, etc.

In this work, we analyze evolution of free volume in glassy 80GeSe_2_-20Ga_2_Se_3_ caused by crystallization treatment at relatively low temperatures (so-called 'cold' crystallization) using combined PAL-DBAL, as well as XRD, AFM, and SEM measuring probes.

## Methods

The ChG of 80GeSe_2_-20Ga_2_Se_3_ composition was prepared from melting mixture of highly pure raw materials (Ge, Ga, and Se of 99.999% purity) in a sealed silica ampoule kept under 10^−6^ Pa vacuum [5,18]. The ampoule of 9-mm inner diameter was placed in a rocking furnace. The raw materials were heated from 20 to 850°C using 2°C/min rate and maintained at this temperature for 12 h at least. Then, the silica tube was quenched in water, annealed at 30°C below glass transition temperature (*T*_g_ = 370°C) for 3 h to minimize inner strains, and slowly cooled down to room temperature. The obtained glass rods were cut into slices of 1 mm in thickness and polished for further optical measurements.

The 'cold' crystallization of 80GeSe_2_-20Ga_2_Se_3_ glass was performed with a single step of thermal treatment at (*T*_g_ + 10)°C. This temperature was chosen as an optimal one for ceramization allowing control simultaneous nucleation and growth of nanoparticles within a glassy matrix in dependence on heat treatment duration. Thus, the glass samples were placed in a ventilated furnace for different times varying from 10 to 100 h, the temperature being kept with an accuracy of ±2°C.

The PAL spectra were recorded with fast coincidence system ORTEC of 230 ps resolution (the full width at half maximum (FWHM) of a single Gaussian determined by measuring ^60^Co isotope) at the temperature of *T* = 22°C and relative humidity of RH = 35%, provided by special climatic installation [18-20]. Two identical samples were used to build *a sandwich geometry* needed for PAL measurements. Two independent PAL experiments were assembled with each sample of the same thermal prehistory, the obtained results agreeing well with each other within an experimental error bar. Each PAL spectrum was measured with a channel width of 6.15 ps (the number of channels was 8000) and contained no less than 10^6^ coincidences in total, which can be considered as conditions of improved measurement statistics. The ^22^Na isotope of slight activity (approximately 50 kBq) prepared from aqueous solution of ^22^NaCl wrapped by Kapton® foil of 12-μm thickness and sealed was used as a source of positrons.

The measured PAL spectra were processed with LT 9.0 program [21]. In our previous work [18], we applied a two-component fitting procedure to reconstruct the measured PAL spectra, this being achieved by corresponding choice of source contribution (nearly 17% in short- and 2% in long-lived source components). The improved statistical treatment in this research for a majority of the studied samples testifies that three-component unconstrained fitting has an obvious preference in view of better goodness for PAL spectra accumulated under solely source contribution (15% in short-lived source component). Thus, the best results were obtained using three discrete components with *τ*_*1*_, *τ*_*2*_, and *τ*_*3*_ lifetimes and *I*_*1*_, *I*_*2*_, and *I*_*3*_ intensities. Despite eventual channel of Ps decaying (with only slight intensity not exceeding 3%) under such treatment, this procedure did not introduce significant changes in the positron trapping modes (e.g., average positron lifetimes *τ*_av_, positron lifetime in defect-free bulk *τ*_b_, positron trapping rate in defects *κ*_d_, and fraction of trapped positrons *η*) calculated using a formalism of two-state positron trapping model [13,14,18-25]. The resulting inaccuracies in positron lifetimes *τ* and intensities *I* were ±0.003 ns and ±0.01 au, respectively, which led to ±0.01 ns^−1^ error bar in positron trapping rate of defects *κ*_d_.

A strict analysis of positron annihilation inputs, however, strongly depends on correct understanding of defect-free positron lifetime *τ*_b_ nature, especially in case of complicated compositional trends associated with significant changes in the type of glass-forming structural units [14]. Since general procedure of PAL spectra treatment includes some uncertainties [26], the proper data processing algorithm should be developed to unambiguously compare physically real annihilation channels and mathematically fitted components. Nevertheless, for the present analysis, we have explored only a simplified approach based on appropriate *error analysis* of PAL data and *background removal*. In addition, the *(τ*_2_*-τ*_b_*)* difference was accepted as size measure for extended free-volume defects where positrons are trapped (in terms of equivalent number of monovacancies), as well as the *τ*_2_*/τ*_b_ ratio was taken in a direct correlation to the nature of these defects [13,14].

The experimental system used for DBAR measurements was arranged like in the PAL geometry using high-purity HP Ge detector with energy resolution of 1.54 at 511 keV. The calibration of multichannel analyzer was performed with set of standard radioactive sources having high-resolved γ-photopeaks: the ^214^Pb isotope with γ photopeaks at 241.92 keV (FWHM = 1.54 keV), 295.21 keV (FWHM = 1.53 keV), 351.92 (FWHM = 1.52 keV), and ^214^Bi isotope having γ-photopeak on the right hand from the analyzed positron-electron annihilation line (511 keV) at 609.31 keV (FWHM = 1.54 keV). The shape of 511-keV annihilation line obtained for studied samples was analyzed by determining so-called *S* and *W* parameters [27]. The *S* parameter defined as a ratio of counts in the central part to the total area of the annihilation line characterizes annihilation of positrons with low-momentum valence electrons in a sample (this parameter is sensitive to free-volume defects). The *W* parameter defined as a ratio of counts in the wing parts to the total area of the annihilation line corresponds to annihilation of positrons with high-momentum core electrons (this parameter is more sensitive to chemical surrounding at the annihilation site) [28]. For Doppler broadening spectra, the energy range of *S-W* parameterization was chosen from 502.29 to 519.71 keV (Δ*E* = 17.42 keV), which corresponds to 260 channels, thus giving overall energetic resolution of 0.067 keV/channel. Two independent measurements consisting of approximately 2 ⋅ 10^6^ counts were performed for each sample to reproduce the analyzed DBAL spectrum. The relative errors in *S* and *W* parameters determined under such measuring protocol (when studied samples affected by different thermal treatments were removed from apparatus during measurement or principally different samples were probed) were 0.3 and 1.5%, respectively [27,28]. Since *S* parameter was chosen to be near a reference value of approximately 0.5 in DBAL measurements [13,16,27,28], it could not be determined better than ±0.0015.

The XRD measurements with CuK_α1_ radiation were performed to determine crystalline phases in the studied samples. Solid-rock plates of powdered 80GeSe_2_-20Ga_2_Se_3_ ChG deposited on amorphous substrate were prepared to arrange experiments in optimal transmittance geometry. The measured X-ray beam intensities and reflection angles 2*θ* were obtained using automatic STOE STADI P diffractometer (STOE & Cie GmbH, Darmstadt, Germany) with a linear position-precision detector. Experimental linear absorption coefficients were determined as logarithmic ratio of primary beam intensities after passing through background and studied samples. All measurements were conducted in 2*θ-*step regime, the profiles of peaks being refined using WinPLOTR software [29].

The surface morphology of crystallized 80GeSe_2_-20Ga_2_Se_3_ ChG annealed for 80 h was studied by SEM using a RЕММА-102-02 microscope (SELMI, Sumy, Ukraine). The scanning of sample surface was performed by electron beam with energy of 15 and 20 kV and a diameter of 5 nm in the secondary electron image regime. To prevent charging during SEM cycling, the sample was covered by thin graphite layer transparent for electron beam. In addition, the surface morphology of the glasses annealed at 25 and 80 h was studied by means of Solver P47-PRO AFM, the obtained images being processed with image analysis program (NT-MDT).

Optical transmission spectra were measured by Shimadzu UV-3600 spectrophotometer operated at room temperature in a spectral region of 600 to 1600 nm.

## Results and discussion

**Figure 1:**
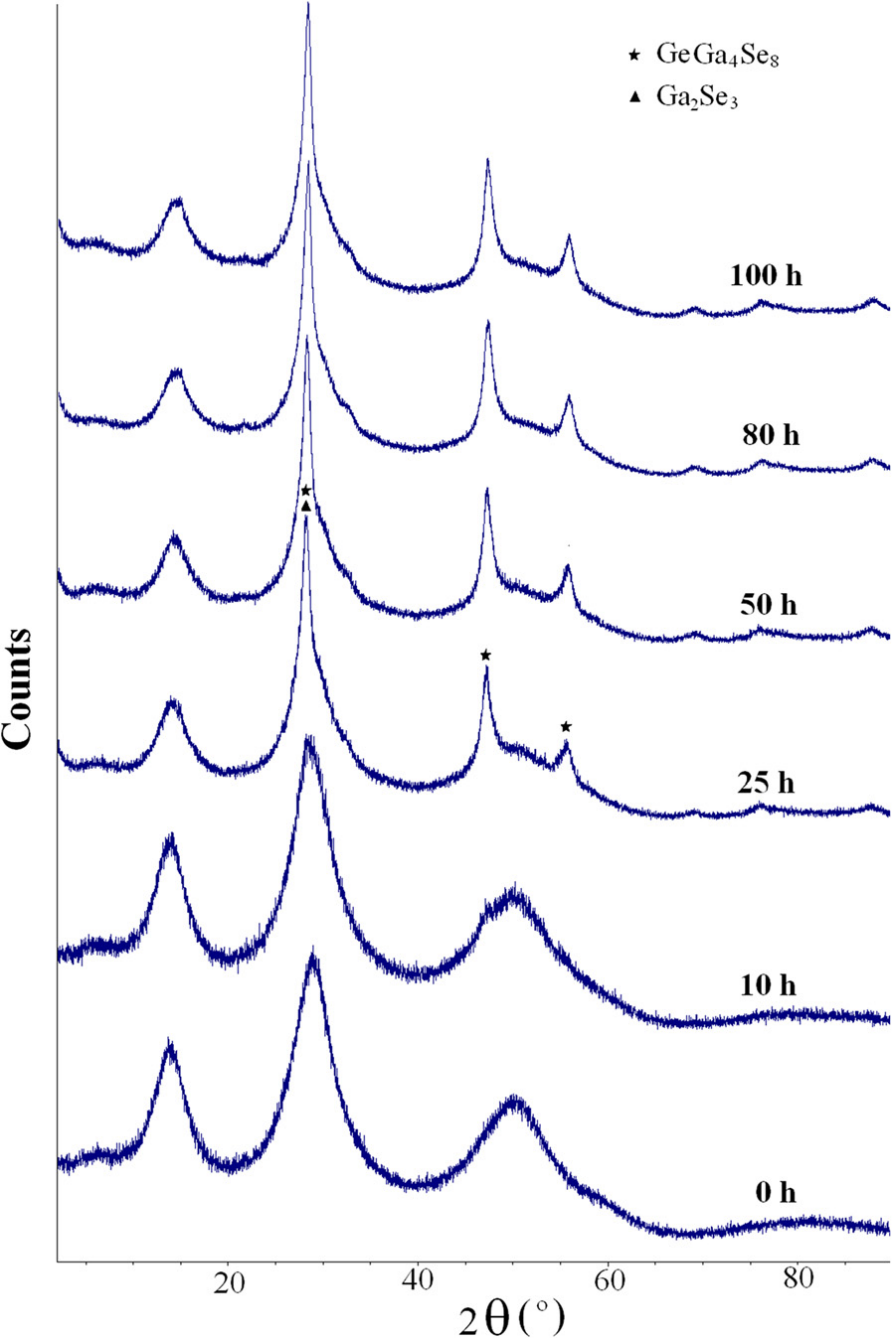
**XRD patterns of 80GeSe**_2_ **-20Ga**_2_ **Se**_3_ **glass before annealing (0 h) and annealed at 380°C for 10, 25, 50, 80, and 100 h.**

The results of XRD measurements for 80GeSe_2_-20Ga_2_Se_3_ glasses before and after thermal annealing at 380°C during different time periods (for 10, 25, 50, 80, and 100 h) are shown in Figure 1. As it was noted earlier [18,22], the annealing at 380°C for 10 h does not change significantly the structure of the ChG, since no sharp crystalline features appear in their XRD patterns apart from noticeable decrease in characteristic amorphous gallous. Therefore, we can assume that even under this short-term heat treatment, there are some transformations in the intrinsic structure of ChG, which do not contribute directly to the crystallization.

With increasing annealing time from 10 to 25, 50 h and further to 80 and 100 h, the well-pronounced crystalline peaks at 2*θ* ~ 28° appear (see Figure 1). The positions of these peaks are in good agreement with GeGa_4_Se_8_ and Ga_2_Se_3_ phase indexation [18], both phases having sharp reflexes near 2*θ* ~ 28°, which cannot be well separated [30]. In fact, all principal XRD peaks of GeGa_4_Se_8_ and Ga_2_Se_3_ phases coincide, so we consider them in crystallized 80GeSe_2_-20Ga_2_Se_3_ glass as signatures of both these phases (Ga_2_Se_3_ and GeGa_4_Se_8_). The width of this peak (2*θ* ~ 28°) confirms the presence of dispersed nanoparticles in a glassy matrix in form of nanocrystalline inclusions of 9 to 10 nm in sizes (determined in respect to the Debay-Scherrer equation [31]) which is in good agreement with previous results [1,6,32-34]. It should be underlined that the height of this peak in glasses annealed at 80 and 100 h does not change essentially in comparison with ChG treated at 50 h. Such behavior testifies in a favor of saturated crystallization at longer durations of annealing.

**Figure 2:**
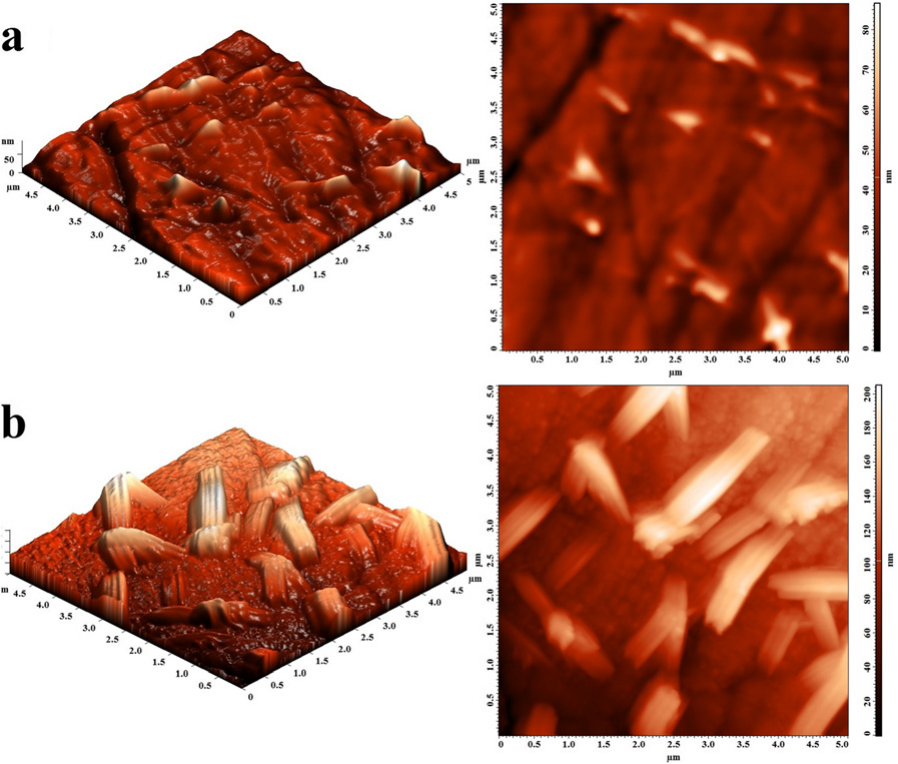
**AFM images of 80GeSe**_2_ **-20Ga**_2_ **Se**_3_ **glass annealed at 380°C for 25 (a) and 80 (b) h.**

**Figure 3:**
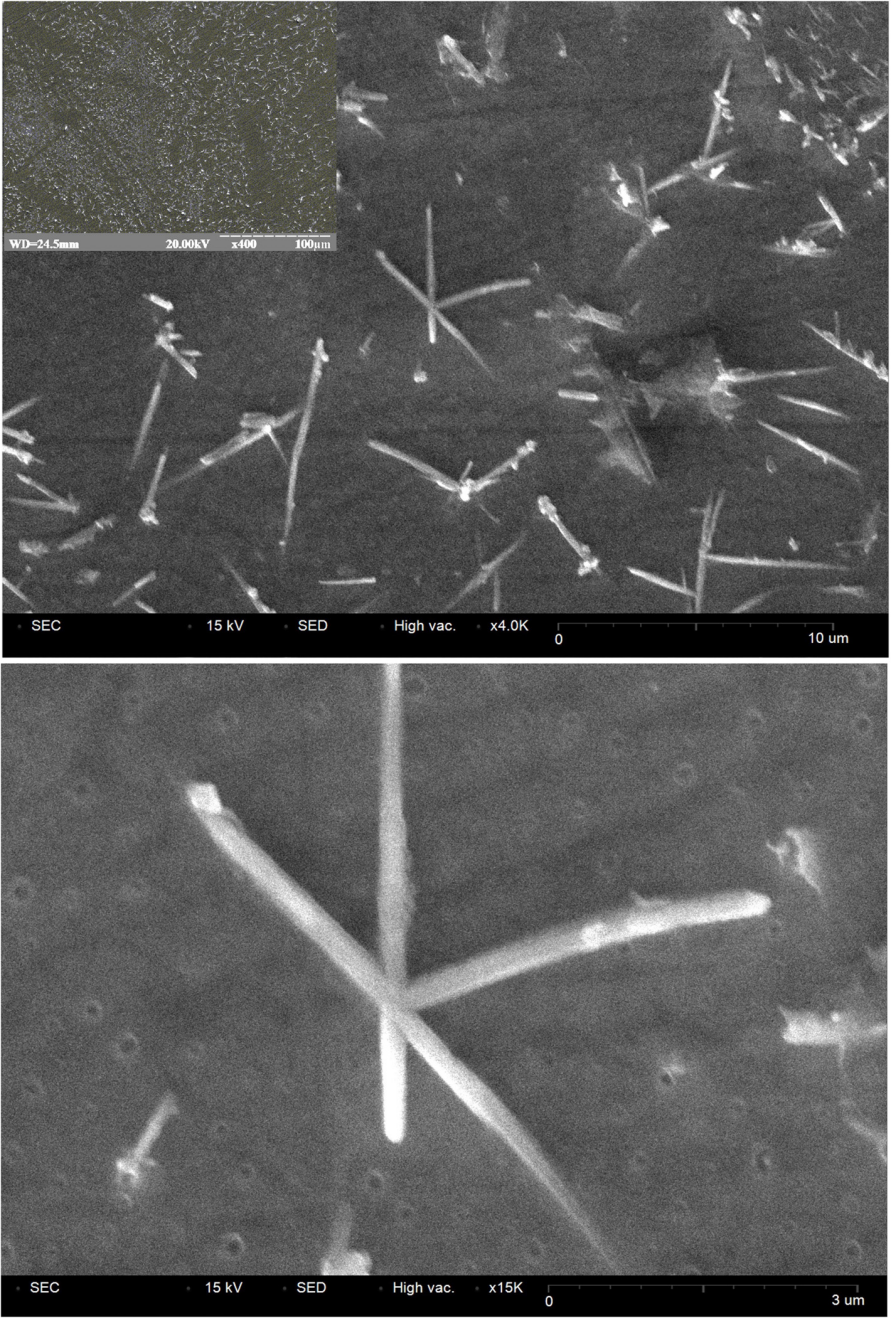
**SEM images of 80GeSe**_2_ **-20Ga**_2_ **Se**_3_ **glass annealed at 380°C for 80 h.**

The maxima associated with GeSe_2_ phase appear on the XRD patterns of thermally annealed 80GeSe_2_-20Ga_2_Se_3_ glass too [18], but (in contrast to [1]) they cannot be well distinguished as separate crystalline peaks even for prolonged annealing. It means that GeSe_2_ crystals appear only in a small amount. However, after longer treatment (over 50 h), surface crystallization occurs more efficiently. To better understand these processes, the ChG annealed for 25 and 80 h at 380°C were examined by AFM and SEM. As shown in Figure 2a, crystallization of GeSe_2_ phase in samples annealed for 25 h begins on a surface. With annealing increased to 80 h, the GeSe_2_ crystals in form of wires with 1- to 3-μm lengths are non-uniformly distributed on sample surface (see Figures 2b and 3).

**Figure 4:**
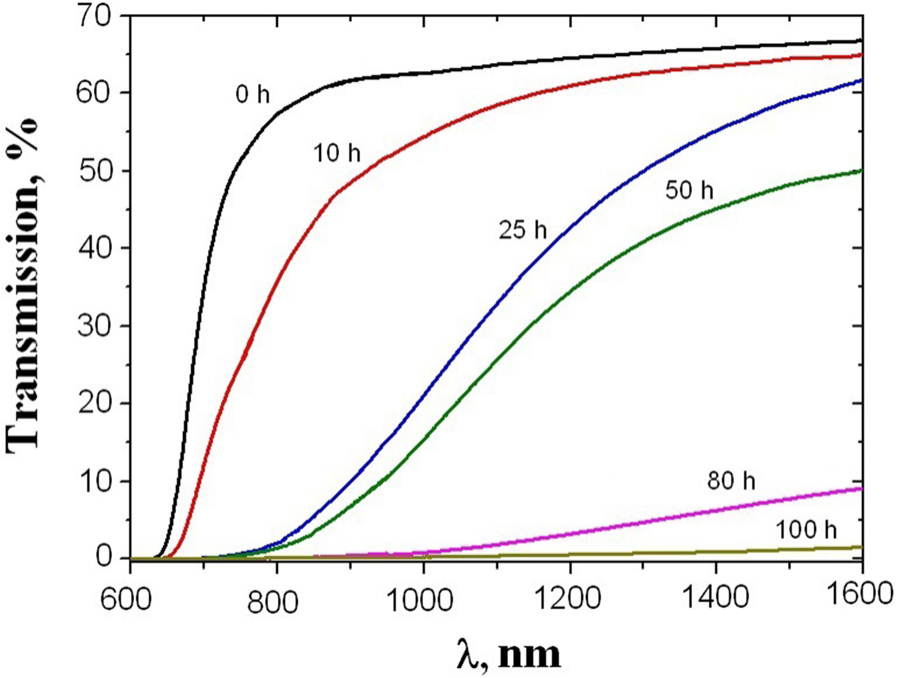
**Transmission in the near-IR for 80GeSe**_2_ **-20Ga**_2_ **Se**_3_ **glass after different heat treatment times at 380°C.**

The process of cold crystallization in 80GeSe_2_-20Ga_2_Se_3_ glasses influences their optical transmission spectra. The non-annealed glassy samples show maximum optical transmittance at the level of 65% (Figure 4). Annealing at 380°C decreases this transmittance and shifts optical transmission edge in a long-wave side. The appearance of growing of Ga_2_Se_3_ and GeGa_4_Se_8_ nanocrystals inside glassy matrix induces light scattering at shorter wavelengths. With increasing heat treatment to 80 and 100 h, the crystallization of GeSe_2_ on glass surface provokes decrease in optical transmittance. Generally, this phenomenon shows the presence of large crystals that deteriorate optical transparency of the material rapidly, leading progressively to its whole opacity in IR range [1]. It can be concluded that large GeSe_2_ crystallites are precipitated on the surface of glasses crystallized for a long time. The sizes of inner Ga_2_Se_3_ and GeGa_4_Se_8_ nanocrystallites are much smaller than those of GeSe_2_ crystals on the surface and do not change with heat treatment above 50 h. It means that new Ga_2_Se_3_ and GeGa_4_Se_8_ nanocrystallites do not appear in a bulk under prolonged annealing, while void fragmentation further proceeds in thermally-relaxed glassy matrix.

Therefore, significant changes in the atomistic structure of 80GeSe_2_-20Ga_2_Se_3_ glasses from disordered amorphous to crystallized one are accompanied by corresponding transformations in the atomic-deficient void structure, the latter being defined by modifications in a void geometry [28]. The results of combined PAL-DBAL measurements below confirm such diversity of void evolution processes in the studied glasses. Best-fit positron trapping parameters along with error bars of three-component fitting parameters and corresponding positron trapping modes for PAL spectra of 80GeSe_2_-20Ga_2_Se_3_ glasses are given in Tables 1 and 2, respectively. Assuming two-state positron trapping model for ChG [13,14,16], two components in the fit of experimental PAL spectra can be associated with annihilation from defect-free bulk states and one type of positron-trapping defects. The *τ*_2_ lifetime is directly related to size of free-volume entities (trapping centers), and intensity *I*_2_ is proportional to the number of such 'defects' under condition of the same defect-free bulk annihilation lifetime [14,25]. The third (*τ*_3_, *I*_3_) component (*τ*_3_, *I*_3_) in the envelope of the fitting curves corresponds to Ps formation on level of 3%. So, we will focus our further analysis on the second (*τ*_2_, *I*_2_) component alone.

**Table 1:** **Fitting parameters for PAL spectra of 80GeSe**_2_ **-20Ga**_2_ **Se**_3_ **glasses before and after thermal annealing**

**Treatment conditions**	**Fitting parameters**					
	***τ***_1_ **, ns**	***I***_1ua_ **, %**	***τ***_2_ **, ns**	***I***_2au_ **, %**	***τ***_3_ **, ns**	***I***_3au_ **, %**
Untreated, 0 h	0.209	0.610	0.426	0.360	1.967	0.030
380°C, 10 h	0.214	0.618	0.428	0.351	2.059	0.031
380°C, 25 h	0.215	0.633	0.432	0.337	2.038	0.030
380°C, 50 h	0.210	0.605	0.424	0.365	2.159	0.030
380°C, 80 h	0.208	0.580	0.415	0.389	2.131	0.031
380°C, 100 h	0.206	0.553	0.403	0.416	1.988	0.031

**Table 2:** **Positron trapping modes for PAL spectra of 80GeSe**_2_ **-20Ga**_2_ **Se**_3_ **glasses before and after thermal annealing**

**Treatment conditions**	**Positron trapping modes**					
	***τ***_av_ **, ns**	***τ***_b_ **, ns**	***κ***_d_ **, ns**	***τ***_2_ **-** ***τ***_b_ **, ns**	***τ***_2_ **/** ***τ***_b_	***η***
Untreated, 0 h	0.290	0.258	0.91	0.17	1.65	0.19
380°C, 10 h	0.291	0.261	0.84	0.17	1.64	0.18
380°C, 25 h	0.291	0.261	0.82	0.17	1.66	0.18
380°C, 50 h	0.292	0.260	0.92	0.16	1.63	0.19
380°C, 80 h	0.283	0.260	0.96	0.16	1.60	0.20
380°C, 100 h	0.291	0.261	1.03	0.14	1.54	0.21

**Figure 5:**
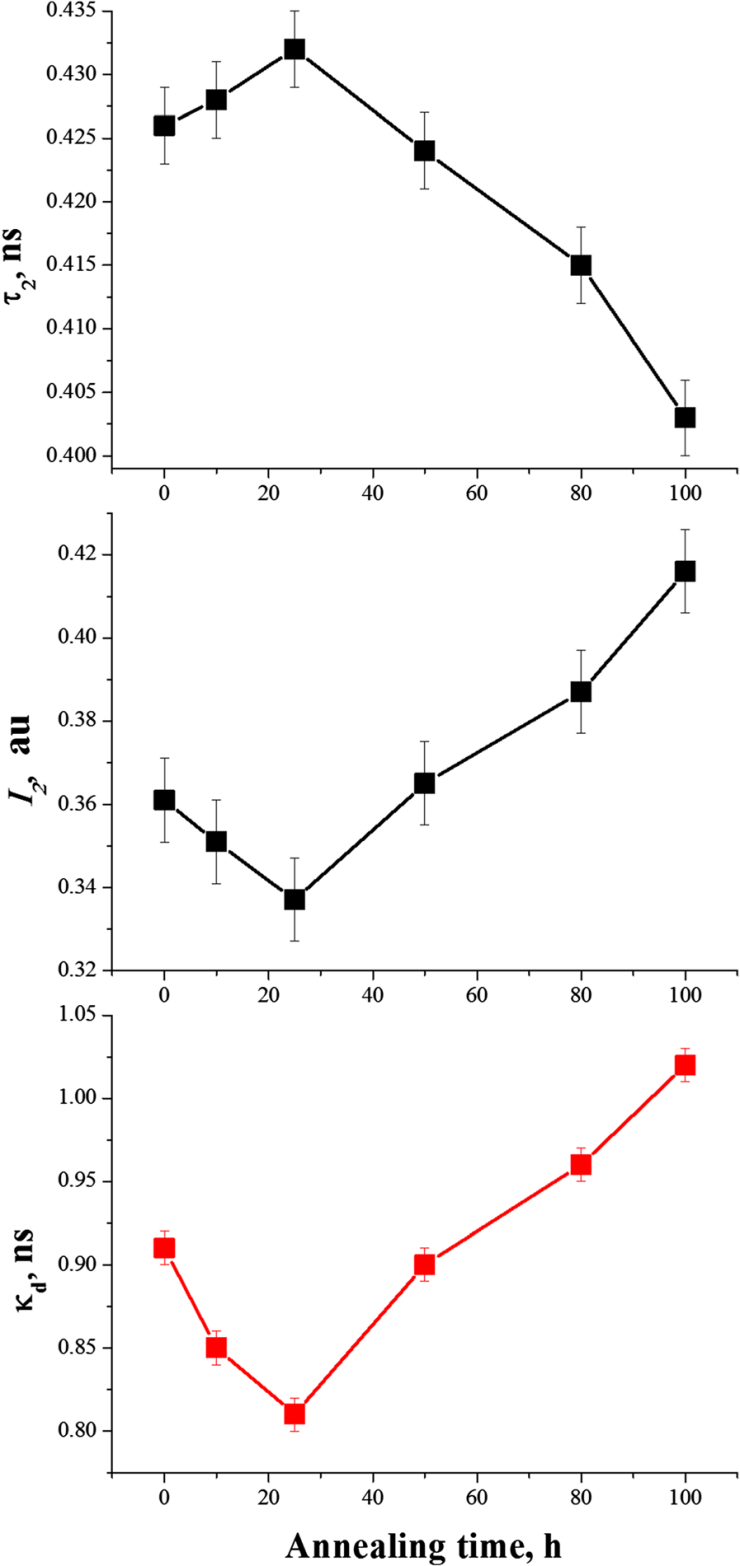
**Defect-related component and positron trapping rate in defects as a function of annealing time in 80GeSe**_2_ **-20Ga**_2_ **Se**_3_ **glass.**

In order to clarify a correlation of the measured positron lifetimes with crystallization behavior and void formation in 80GeSe_2_-20Ga_2_Se_3_ glass as result of thermal annealing, the parameters of defect-related component (first of all, the positron trapping rate in defects *κ*_d_) are plotted as a function of thermal annealing time (Figure 5). With increase in the annealing duration to 10 and 25 h, the lifetime *τ*_2_ increases and *I*_2_ intensity decreases due to void expansion and agglomeration. This trend correspondingly reduces the positron trapping rate *κ*_d_ without significant changes in *τ*_av_ and *τ*_b_ lifetimes. With further annealing duration proceeding to 50, 80, and 100 h, the *I*_2_ intensity ceases to increase, while lifetime *τ*_2_ appreciably decreases to 0.424, 0.415, and 0.403 ns, respectively. These changes result in increased positron trapping rate *κ*_d_. Other positron trapping parameters such as *τ*_2_/*τ*_b_ and *η* behave under annealing in a line with these changes (Table 2), but *(τ*_2_*-τ*_b_*)* difference, which can be accepted as a size measure for extended free-volume defects where positrons are trapped [13], decreases with annealing duration. The fraction of trapped positrons *η* decreases in the initial stage of treatment to 25 h and increases at further annealing from 50 to 100 h.

During crystallization, the glass structure relaxes towards more thermodynamically favorable state, which can be characterized by gain in configurational entropy, enthalpy, or free volume [14]. It means that existing free-volume voids can be essentially transformed under this process, giving a resulting shrinkage in the atomic structure. The fragmentation of larger free-volume entities into smaller ones occurs like as during natural physical ageing [35-37]. Such process is accompanied by decrease in *τ*_2_ lifetime and corresponding increase in *I*_2_ intensity.

In contrast to the above fitting PAL parameters (*τ*_1_, *I*_1_, *τ*_2_, *I*_2_), the changes in the positron trapping rate of free-volume defects *κ*_d_ are more pronounced, especially at longer annealing durations, when specific fragmentation reveals decrease in dimensions of these defects accompanied by simultaneous increase in their amount (see Table 2 and Figure 5). In principle, these changes in *κ*_d_ can be caused by charge state of trapping centers too [15]. However, the constant *τ*_2_/*τ*_b_ ratio close to 1.6 for all ChG samples, despite their treatment duration, testified that corresponding positron-trapping centers are rather of the same type, being most probably as large as di- or tri-atomic vacancies [18].

**Figure 6:**
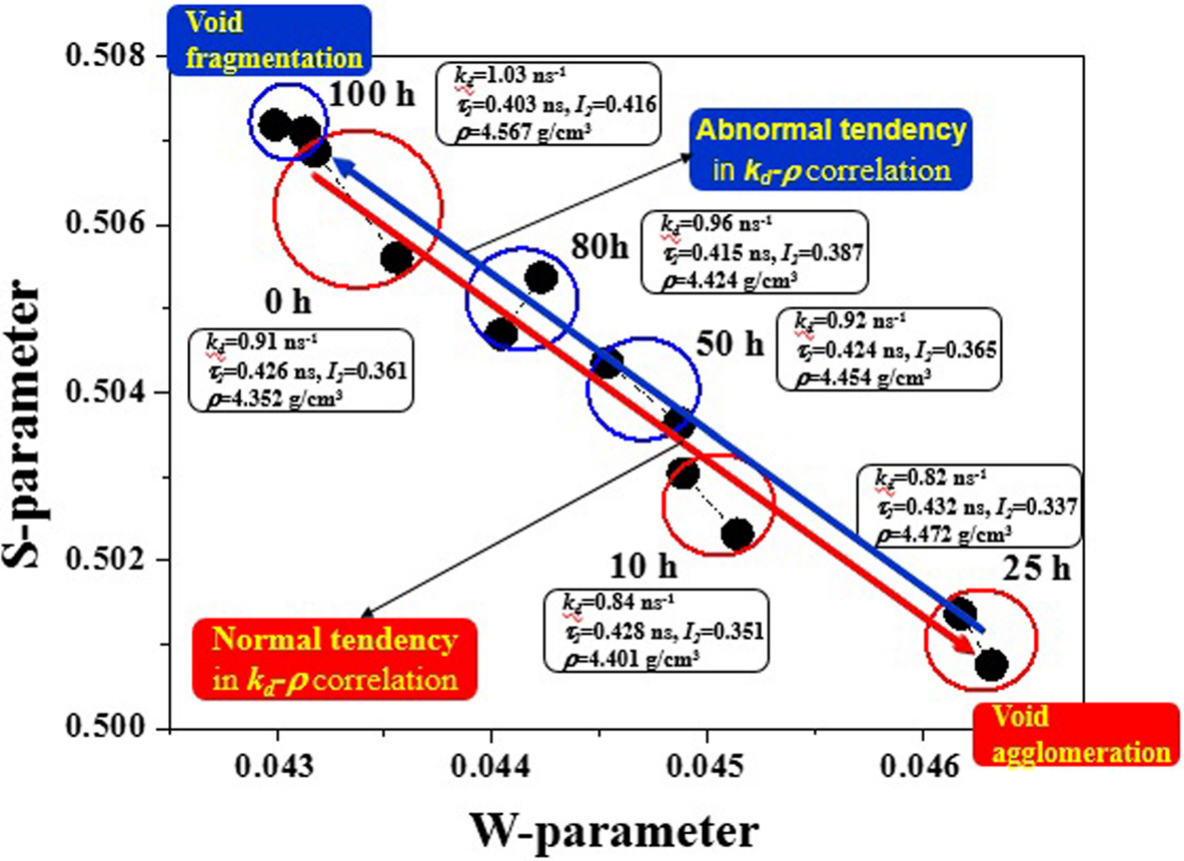
***S*** **-** ***W*** **correlation plot obtained with DBAR technique for 80GeSe**_2_ **-20Ga**_2_ **Se**_3_ **glass.**

These findings are well supported by the results of DBAL measurements presented in the form of *S-W* correlation plot in Figure 6. It is shown that all points on this plot are grouped along nearly the same straight line trajectory tending from initial glass (with atomic density of *ρ* = 4.352 g/cm^3^) to partially crystallized one annealed at 25 h (*ρ* = 4.472 g/cm^3^) in the direction of reduced *S* and enhanced *W* parameters. Such behavior corresponds to a so-called normal tendency in the *S-W* evolution [28], when overall free volume of positron trapping voids is mainly responsible for atomic density of the samples. Normal tendency in *κ*_d_*-ρ* correlation reflects void agglomeration in the initial stage of thermal annealing for 10 and 25 h. By accepting that defect-free bulk positron lifetime *τ*_b_ is almost not affected by crystallization in these glasses (as it really follows from Table 2), this result speaks in favor of unchanged nature of corresponding free-volume voids responsible for positron trapping, when only concentration of these traps is a subject to most significant changes. At further annealing of 80GeSe_2_-20Ga_2_Se_3_ ChG for 50, 80, and 100 h, the *S-W* evolution changes to a so-called abnormal tendency in *κ*_d_*-ρ* correlation reflecting the process of void fragmentation (the exception constitutes only the sample annealed for 100 h because of possible macroscopic imperfections).

Thus, nearly the same *τ*_b_ and *τ*_av_ values are characteristic for all ChG samples, while the positron trapping rate in extended defects *κ*_d_ increases with nucleation of crystallized phases and decreases with further crystallization (Table 2). It means that the same type of free-volume voids governs positron annihilation in the studied glasses affected to cold crystallization. The crystal growth is accompanied by network shrinkage of 80GeSe_2_-20Ga_2_Se_3_ glass, the agglomeration of free-volume voids occurring more rapidly than their appearance due to mismatch between growing crystallites and remainder of the glass matrix.

## Conclusions

Cold crystallization behavior of 80GeSe_2_-20Ga_2_Se_3_ glasses during annealing at 380°C for 10, 25, 50, 80, and 100 h indicates the formation of GeGa_4_Se_8_ and Ga_2_Se_3_ crystallizes in nanoparticle form in the inner structure of these glasses. The accompanying crystallization of GeSe_2_ phase is mainly a surface-related phenomenon for prolonged heat treatments. The continuing crystal evolution with duration of thermal annealing is revealed by decrease in optical transmittance and long-wave shift in optical transmission edge of the studied glasses. The cold crystallization is shown to be associated with specific fragmentation of larger free-volume entities (acting as positron trapping voids) into a greater number of smaller ones. This process can be presented as abnormal tendency in the correlation between positron trapping rate *κ*_d_ in defects and atomic density of the crystallized material *ρ*. The initiating stage of the crystallization occurring under 10 and 25 h annealing has an opposite nature, demonstrating a preferential crystallites nucleation and agglomeration of related voids with normal tendency in *κ*_d_*-ρ* correlation.

## References

[1] Roze M, Calvez L, Ledemi Y, Allix M, Matzen G, Zhang XH (2008). Optical and mechanical properties of glasses and glass–ceramics based on the Ge–Ga–Se system. J Am Ceram Soc..

[2] Sakai T, Maeda K, Munzar M, Tonchev D, Ikari T, Kasap SO (2006). Thermal and optical analysis of Ge–Ga–Se chalcogenide glasses. Phys Chem Glasses..

[3] Calvez L, Ma HL, Lucas J, Zhang XH (2007). Selenium-based glasses and glass ceramics transmitting light from the visible to the far-IR. Adv Mater..

[4] Petkova T, Nedevaa Y, Petkova P (2001). Compositional trends of the properties in chalcogenide Ge-Se-Ga glasses. J Optoelectronics Adv Mater..

[5] Calvez L, Lin C, Rozé M, Ledemi Y, Guillevic E, Bureau B (2010). Similar behaviors of sulfide and selenide-based chalcogenide glasses to form glass-ceramics. Proc of SPIE.

[6] Calvez L, Roze M, Ledemi Y, Ma H-L, Lucas J, Allix M (2008). Controlled crystallization in Ge-(Sb/Ga)-(S/Se)-MX glasses for infrared applications. J Ceramic Soc Japan..

[7] Savchyn P, Karbovnyk I, Vistovskyy V, Voloshinovskii A, Pankratov V, Cestelli Guidi M (2012). Vibrational properties of LaPO_4_ nanoparticles in mid- and far-infrared domain. J Appl Phys.

[8] Baran J, Pasechnik YA, Shportko KV, Trzebiatowska-Gusowska M, Venger EF (2006). Raman and FIR reflection spectroscopy of ZnP_2_ and CdP_2_ single crystals. J Mol Struct..

[9] Karbovnyk I, Lesivtsiv V, Bolesta I, Velgosh S, Rovetsky I, Pankratov V (2013). BiI_3_ nanoclusters in melt-grown CdI_2_ crystals studied by optical absorption spectroscopy. Phys B Condens Matter..

[10] Shportko KV, Pasechnik YA, Wuttig M, Rückamp R, Trukhan VM, Haliakevich TV (2009). Plasmon-phonon contribution in the permittivity of ZnP_2_ single crystals in FIR at low temperatures. Vib Spectros..

[11] Huczko A, Dabrowska A, Savchyn V, Popov AI, Karbovnyk I (2009). Silicon carbide nanowires: synthesis and cathodoluminescence. Physica Status Solidi..

[12] Voloshynovskii A, Savchyn P, Karbovnyk I, Myagkota S, Cestelli Guidi M, Piccinini M (2009). CsPbCl_3_ nanocrystals dispersed in the Rb_0.8_Cs_0.2_Cl matrix studied by far-infrared spectroscopy. Solid State Comm.

[13] Krause-Rehberg R, Leipner H (1999). Positron annihilation in semiconductors: defect studies.

[14] Shpotyuk O, Filipecki J (2003). Free volume in vitreous chalcogenide semiconductors: possibilities of positron annihilation lifetime study.

[15] Dlubek G, Yang Y, Krause-Rehberg R, Beichel W, Bulut S, Pogodina N (2010). Free volume in imidazolium triflimide ([C3MIM][NTf2]) ionic liquid from positron lifetime: amorphous, crystalline, and liquid states. J Chem Phys.

[16] Jean YC, Mallon PE, Schrader DM (2003). Principles and application of positron and positronium chemistry.

[17] Sabelová V, Petriska V, Veterníková J, Slugeň V, Degmová J, Kilpeläinen S (2013). Defect detection in Fe-Cr alloys with positron annihilation doppler broadening spectroscopy. Mater Sci Forum..

[18] Shpotyuk O, Calvez L, Petracovschi E, Klym H, Ingram A, Demchenko P (2014). Thermally-induced crystallization behaviour of 80GeSe_2_–20Ga_2_Se_3_ glass as probed by combined X-ray diffraction and PAL spectroscopy. J Alloy Comp..

[19] Klym H, Ingram A, Hadzaman I, Shpotyuk O (2014). Evolution of porous structure and free-volume entities in magnesium aluminate spinel ceramics. Ceram Int..

[20] Ingram A, Klym HI, Shpotyuk OI. Ge-Ga-S/Se glasses studied with PALS technique in application to chalcogenide photonics, Proc. of the International Conference on Advanced Optoelectronic and Laser, CAOL. 2013; 386–387.

[21] Kansy J (1996). Microcomputer program for analysis of positron annihilation lifetime spectra, *Nucl*. Instr Methods in Phys Res A..

[22] Klym H, Ingram A, Shpotyuk O. Crystallization processes in Ge-Ga-Se glasses studied with positron annihilation technique. Microelectronics Proceedings-MIEL 2014, 2014 29th International Conference. 2014: 277–278. doi:10.1109/MIEL.2014.6842141

[23] Klym H, Ingram A (2007). Unified model of multichannel positron annihilation in nanoporous magnesium aluminate ceramics. J Phys Conf Ser.

[24] Klym H, Hadzaman I, Ingram A, Shpotyuk O (2014). Multilayer thick-film structures based on spinel ceramics. Can J Phys..

[25] Karbovnyk I, Bolesta I, Rovetski I, Velgosh S, Klym H (2014). Studies of CdI_2_–Bi_3_ microstructures with optical methods, atomic force microscopy and positron annihilation spectroscopy. Mater Sci-Poland.

[26] Nambissan PMG, Upadhyay C, Verma HC (2003). Positron lifetime spectroscopic studies of nanocrystalline ZnFe_2_O_4_. J Appl Phys..

[27] Liszkay L, Corbel C, Baroux L, Hautojarvi P, Bayhan M, Brinkman W (1994). Positron trapping at divacancies in thin polycrystalline CdTe films deposited on glass. Phys Lett..

[28] Shpotyuk O, Ingram A, Bureau B, Shpotyuk Y, Boussard-Pledel C, Nazabal V (2014). Positron annihilation probing of crystallization effects in TAS-235 glass affected by Ga additions. J Phys Chem Solid..

[29] Roisnel T, Rodriguez-Carvajal J (2001). WinPLOTR: a Windows tool for powder diffraction patterns analysis. Mater Sci Forum..

[30] Loireau Lozac'h AM, Guittard M (1977). Système ternaire La_2_Se_3_-Ga_2_Se_3_-GeSe_2_ Diagramme de phase - Etude des verres. Mater Res Bull..

[31] Keshari AK, Pandey AC (2008). Size and distribution: a comparison of XRD, SAXS and SANS study of II–VI semiconductor nanocrystals. J Nanoscince Nanotechnology..

[32] Lin C, Calvez L, Rozé M, Tao H, Zhang X, Zhao X (2009). Crystallization behavior of 80GeS_2_-20Ga_2_S_3_ chalcogenide glass. Appl Phys A..

[33] Lin C, Calvez L, Tao H, Allix M, More A, Zhang X (2011). Evidence of network demixing in GeS_2_–Ga_2_S_3_ chalcogenide glasses: a phase transformation study. J Solid State Chem..

[34] Mosselin P, Le Coq D, Calvez L, Petracovschi E, Lepine E, Bychkov E (2012). CsCl effect on the optical properties of the 80GeS_2_–20Ga_2_S_3_ base glass. Appl Phys A..

[35] Golovchak R, Ingram A, Kozyukhin S, Shpotyuk O (2013). Free volume fragmentation in glassy chalcogenides during natural physical ageing as probed by PAL spectroscopy. J Non-Cryst Sol..

[36] Golovchak R, Kozdras A, Balitska V, Shpotyuk O (2012). Step-wise kinetics of natural physical ageing in arsenic selenide glasses. J Phys Condens Matter.

[37] Ingram A, Golovchak R, Kostrzewa M, Wacke S, Shpotyuk M, Shpotyuk O (2012). Compositional dependences of average positron lifetime in binary As–S/Se glasses. Physica B..

